# Research about the Characteristics of Chaotic Systems Based on Multi-Scale Entropy

**DOI:** 10.3390/e21070663

**Published:** 2019-07-06

**Authors:** Chunyuan Liu, Lina Ding, Qun Ding

**Affiliations:** 1 Electronic Engineering College, Heilongjiang University, Harbin 150080, China; 2 Computer and Information Engineering College, Heilongjiang University of Science and Technology, Harbin 150027, China; 3 Electrical Engineering College, Suihua University, Suihua 1520061, China

**Keywords:** chaos, multi-scale entropy, the logistic system, variance

## Abstract

The logistic chaotic system, as a classical complex phenomenon of nonlinear dynamic systems, has received extensive attention in the field of secure communication. It is generally believed that the characteristics of chaos are suitable for the needs of encryption systems. In this paper, a multi-scale entropy theory analysis and statistical analysis are carried out on the chaotic sequences produced by different parameters and different initial values of logistic systems. According to the simulation results, the complexity of the chaotic system represented by the logistic system is mainly decided by parameter *μ*. Not all characteristic parameters of the chaotic system depend on the initial values. It is possible to make a reasonable estimation and prediction of the chaotic system from a macroscopic level. A variance estimation method for the parameter *μ* is proposed and applied to a logistic system and to another chaotic system, which is equally effective.

## 1. Introduction

Chaos, as a classical complex phenomenon of a nonlinear dynamic system, is widely used in military and commercial spread spectrum communication systems with high demands for secrecy, because of its characteristics of wideband, quasi-noise, and sensitivity to initial state [1]. The chaotic system, a logistic chaotic map, has been paid much attention by researchers and has been studied extensively. In 1978, Feigenbaum [2] made a detailed analysis of its mathematical properties. The literature [3] has realized the bifurcation control of the multiple periods of the logistic model. The authors of [4] discussed the influence of delay time on the transition probabilities between the metastable state and the stable state of a logistic system. The authors of [5] applied the model to the evolution of biomolecular networks. The authors of [6,7] made a detailed description of the bifurcation and fractional dimension of the multiple complex logistic systems. The authors of [8] studied the chaotic fractal characteristics of C-K mapping, which is partially similar to logistic sequences. It can be seen that the logistic model has had a lot of research done on its local features, and these research work plays an important role in the field of information communication.

As a chaotic system, unpredictability and uncertainty are its most important features. From the point of view of information theory, to study the whole uncertainty of a function, in addition to studying its closed expression, we can also make a statistical analysis and a quantification of the discrete sequences generated by it. In information theory, the best way to quantify uncertainty is entropy. A modified permutation entropy was proposed in literature [9], in order to obtain a quantitative estimate of the Kolmogorov-Sinai entropy in hyper-chaotic models. The authors of [10] introduced a multivariate permutation entropy (MvPE) method and used it to quantify the complexity of chaotic systems. As an application, MvPE was applied to analyze the complexity of chaotic systems, including a hyper-chaotic Hénon map, fractional-order simplified Lorenz system, and financial chaotic system. A multivariate multi-scale distribution entropy (MMSDE) was also presented in the literature [11], which was used to assess the complexity of a complex dynamical system. In literature [12,13], randomness and quantifying complexity from information theory were described in detail, and the complexity analysis method was an important measure for the sequences in the stream ciphers. The authors of [14] defined the concept of uniformity through learning the theory of an exclusive sphere, and made a comparative analysis between the Lyapunov exponent and the uniformity of a logistic system, and concluded that the chaotic characteristic of a logistic system is more and more random with the parameter increasing. The greater the randomness of the sequence, the greater the complexity, the higher the entropy, and the greater the difficulty for the sequence to be restored. In recent years, the research on the logistic system model has mainly been focused on its application. The authors of [15] constructed an as-box based on logistic mapping for the advanced encryption standard (AES). The authors of [16] extended further analytical study of the complex dynamics existing in two coupled logistic maps, and utilized it in a suggested real-time text encryption system. However, there are few studies on logistic sequences from a macro perspective, and there is an insufficient understanding of some of the overall characteristics of the system. Only then, will the anti-interference and interception capability of the spread spectrum sequence of the actual secure communication system be affected [17,18,19,20].

This paper applies an MSE algorithm in the complexity analysis of a logistic chaotic system. The distribution characteristics of a logistic chaotic sequence were analyzed from a macroscopic perspective, focusing on the multi-scale entropy distribution characteristics of the system. Moreover, through numerical simulation, it is concluded that the multi-scale entropy of the system is basically determined by the parameter *μ,* and the dependence of the multi-scale entropy of the system on the initial value is not obvious, and the multi-scale entropy of the system also increases with the increase of parameter *μ*. In view of the logistic system, the definite expression of a chaotic system obtains the uncertain sequence, and the uncertain sequence implies certain definite components on the whole. In addition, on the basis of a large number of statistical data, a parameter-*μ* estimation method for the logistic chaotic system is proposed, and the statistical table of the data estimation is obtained.

## 2. Features of Logistic Chaotic System

One-dimensional logistic mapping is as follows:

(1) $$x_{n+1}=\mu x_{n}\left(1-x_{n}\right),0\leq \;x\leq 1,0<\mu <4$$

where *x_n_* is the status value of the logistic mapping, and parameter *μ* is the coefficient of the iterative equation. The chaotic phenomenon is induced by adjusting *μ*. Within the range of *μ* ∈ (3.57, 4], the logistic mapping induces the chaotic phenomenon. If the parameter *μ* and the initial value of system *x*_0_ are given, an iterative computation by Equation (1) can obtain the logistic sequence at random lengths. According to the different parameter of *μ*, the following situations take place after substantial iterations:

1. If *μ* ∈ (0,1), the system stability value is 0;
2. If *μ* ∈ (1,3), the system has two stable points *x* = 0 and *x* = 1 − 1/*μ*;
3. If *μ* ∈ (3,3.499), the system has two periodic points, that is, $\;\varepsilon =\frac{1+\mu \pm \sqrt{\left(\mu +1\right)\left(\mu +3\right)}}{2\mu }.$
4. If *μ* ∈ (3.499,3.544), the system has four periodic points;
5. If *μ* ∈ (3.544,3.564), the system has eight periods.

Hereafter, the system generates doubling period bifurcation. After many bifurcations, it is generally believed that when $x_{\infty }=3.570$, the logistic system enters a chaotic state.

## 3. Multi-Scale Entropy (MSE) of Time Sequence

At present, the approximate entropy (ApEn) algorithm proposed by Pincus et al. [21], and the improved approximate entropy algorithm proposed by Richman et al. [22,23,24] (the sample entropy (SampEn) algorithm) are widely used in the complexity measurement algorithm of chaotic sequences. A heaviside function was adopted to measure the similarity between the two, and it was very sensitive to the values of the threshold (*r*) and phase space dimension (*m*). However, SampEn did not calculate its own matching statistics, so SampEn was a measure generated by new information. This was an improvement on ApEn, but a meaningless *ln0* would appear in the case of no template matching. The authors of [25,26] proposed the multi-scale entropy (MSE) algorithm, which can avoid these phenomena to some extent.

An MSE algorithm based on SampEn is used to describe the degree of irregularity of the time series on different scales. The MSE algorithm involves three parameters—*τ, m,* and *r.* Where *τ* is the scale factor, *m* is the embedded dimension, and *r* is the threshold value (also known as the similarity coefficient). Parameter *r* defines the similarity criterion of the comparing vectors. If the absolute difference of any two vector components is greater than *r* × *SD*, the two vector components are different. Otherwise, they are considered as equal. Theoretically, *r* is acceptable between 0 and 1. However, for discrete-time sequences, a higher resolution ratio inevitably needs a lower *r*, and a larger *r* value comes with a lower entropy value. The computational procedures of the logistic sequences are shown, as follows [27]:

1. Set *x*_1_, *x*_2_, *…*, *x_L_* as a discrete-time sequence, including *L* points. Conduct coarse-graining conversion on the original time series, and divide the original time series into non-overlapping windows with a length of *τ*. Calculate the average value of each window, and obtain the new coarse-graining time series. Each new data point is derived from Equation (2), as follows:
  (2) $$y_{j}^{\left(\tau \right)}=\frac{1}{\tau }\overset{j\tau }{\underset{i=\left(j-1\right)\tau +1}{\sum }}x\left(i\right),\;\;\;\;\;\;j=1∽L/\tau .$$
  In Equation (2), *τ* is the scale factor. Each coarse-graining time series has a length of *L*/*τ*. Equation (3) and Equation (4) are the methods for computing the coarse-graining of the time series, in which *τ* = 2 and *τ* = 3. When *τ* is 1, it is the original series.
  (3) $$\begin{array}{c} \begin{array}{cccccccccccccccc} x_{1} & & x_{2} & & x_{3} & & x_{4} & & x_{5} & & x_{6} & \ldots \ldots & x_{i} & & x_{i+1} & \ldots \ldots \\ \ddots & & ⋰ & & \ddots & & ⋰ & & \ddots & & ⋰ & & \ddots & & ⋰ & \\ & y_{1} & & & & y_{2} & & & & y_{3} & & \ldots \ldots & & y_{j} & & \ldots \ldots \end{array},\\ y_{j}=\frac{x_{i}+x_{i+1}}{2}. \end{array}$$
  (4) $$\begin{array}{c} \begin{array}{ccccccccccc} x_{1} & x_{2} & x_{3} & x_{4} & x_{5} & x_{6} & \cdots \cdots & x_{i} & x_{i+1} & x_{i+2} & \cdots \cdots \\ \ddots & \vdots & ⋰ & \ddots & \vdots & ⋰ & & \ddots & \vdots & ⋰ & \\ & y_{1} & & & y_{2} & & \cdots \cdots & & y_{j} & & \cdots \cdots \end{array},\\ y_{j}=\frac{x_{i}+x_{i+1}+x_{i+2}}{3}. \end{array}$$
2. Then, for a different *τ*, compute the SampEnon coarse-graining time series. SampEn originates from ApEn. However, the calculation of the approximate entropy also involves comparing its own data, which may induce certain errors. In order to reduce the errors, in the literature [28], for the given threshold value *r*, Richman calculated the ratio between the number of $d\left[Y^{\left(\tau \right)}\left(i\right),Y^{\left(\tau \right)}\left(j\right)\right]<r$ and the total distance *N* − *m*, denoted as $C_{i}^{\tau ,m}\left(r\right)$.
  (5) $$C_{i}^{\tau ,m}\left(r\right)=\frac{1}{N-m}num\left\{d\left[Y^{\left(\tau \right)}\left(i\right),Y^{\left(\tau \right)}\left(j\right)\right]<r\right\},\;\;\left(i,j=1∼N-m+1\right),\;i\neq j)$$
3. Calculate the average of all points, as follows:
  (6) $$C^{\tau ,m}\left(i\right)={\left(N-m+1\right)}^{-1}\overset{N-m+1}{\underset{i=1}{\sum }}C_{i}^{\tau ,m}\left(r\right)$$
4. Add dimension *m* by 1, repeat procedures 2–3, and obtain $C_{i}^{\tau ,m+1}\left(i\right)$ of scale *τ*, then recalculate the average $C_{i}^{\tau ,m+1}\left(i\right)$.
  In the actual calculation, use SampEn, as follows:
  (7) $$SampEn\left(\tau ,m,r\right)=-\ln\left[\frac{C^{\tau ,m+1}}{C^{\tau ,m}}\right].$$
  MSE is as follows:
  (8) $$MSE=\{\tau |SampEn\left(\tau ,m,r\right)=-\ln\left[C^{\tau ,m+1}\left(r\right)/C^{\tau ,m}\left(r\right)\right]\}.$$

## 4. Macroscopic Characteristics of Logistic Chaotic Sequences

In order to measure the overall characteristics of the logistic chaotic sequence, all of the initial values and parameters (*μ*) that can be obtained for the sequence are taken. A simulation experiment is conducted in MATLAB R2010. Logistic sequences are generated after 10,000 iterations of Equation (1). The system accuracy utilizes the default double precision of MATLAB. In the MSE analysis, parameter *r* is generally 15% of the standard deviation (SD) and remains unchanged at all scales, and does not recalculate for each coarse-graining time series [29]. For SD, in the process of the initial normalization, the variance changed by coarse-graining is related to the time structure of the original time series, so it should be considered by the calculation of entropy. However, initial normalization may also prevent an MSE value of two different time series from being impacted by variance, and the impact comes from the organization of the sequence itself. The difference value of the two adjacent points in the same chaotic sequence is shown in the following Equation (9).

(9) $$\Delta d=x_{n+1}-x_{n}=\mu x_{n}\left(x-x_{n}\right)-x_{n}=\;\mu x_{n}^{2}+\left(\mu -1\right)x_{n}$$

If $x_{n}=\frac{\mu -2}{2\mu }$, that is $\Delta d_{max}=\frac{{\left(\mu -1\right)}^{2}}{4\mu }$, $x_{n+1}=\frac{\mu^{2}-1}{4\mu }$; besides, if $x_{n}=\frac{\mu +1}{2\mu }$,$\;x_{n+1}=\frac{\mu^{2}-1}{4\mu }$; if *x_n_* = 1/2, *x_n_*_+1_ = 1/*μ*. Hence, if $x_{n}\in \left[\frac{\mu -1}{2\mu },\;\frac{\mu +1}{2\mu }\right]\;$(the width of this interval is 1/*μ*), it is mapped to $x_{n+1}\in \left[\frac{\mu^{2}-1}{4\mu },\;\frac{\mu }{4}\right]$ (width of the interval is 1/4*μ*). Thus, it can be seen that after one iteration, previously wide area is mapped to the upper bound zone of the system, and the interval is decreased.

**Figure 1:**
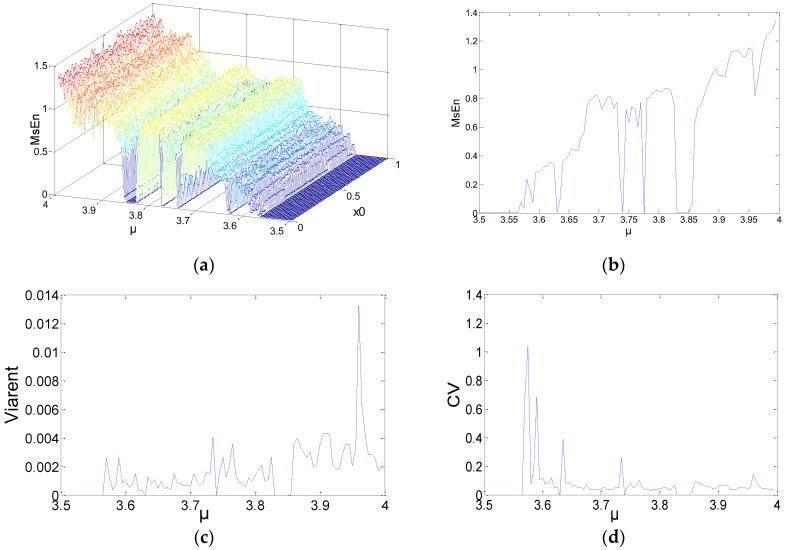
*N* = 1000 multi-scale entropy (MSE) results: (**a**) 3D figure of MSE; (**b**) means of different MSE values; (**c**) variances of MSE; (**d**) coefficient of variable of a different *μ*.

**Figure 2:**
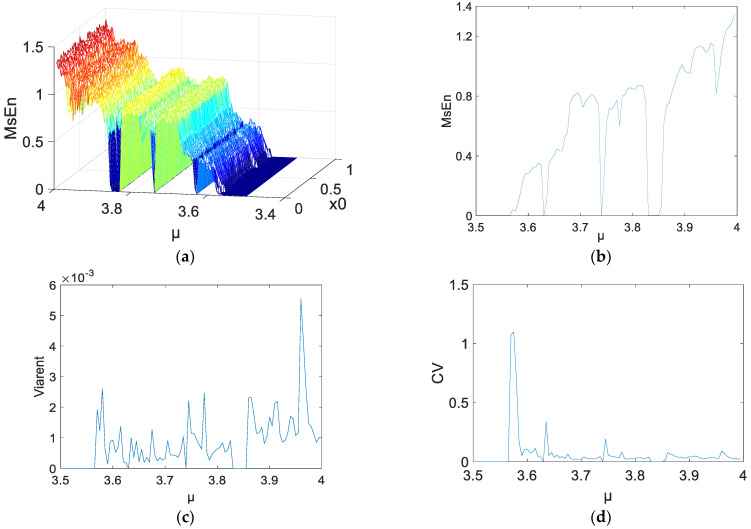
*N* = 2000 MSE results: (**a**) 3D figure of MSE; (**b**) means of MSE; (**c**) variances of MSE; (**d**) coefficient of variable of a different *μ*.

**Figure 3:**
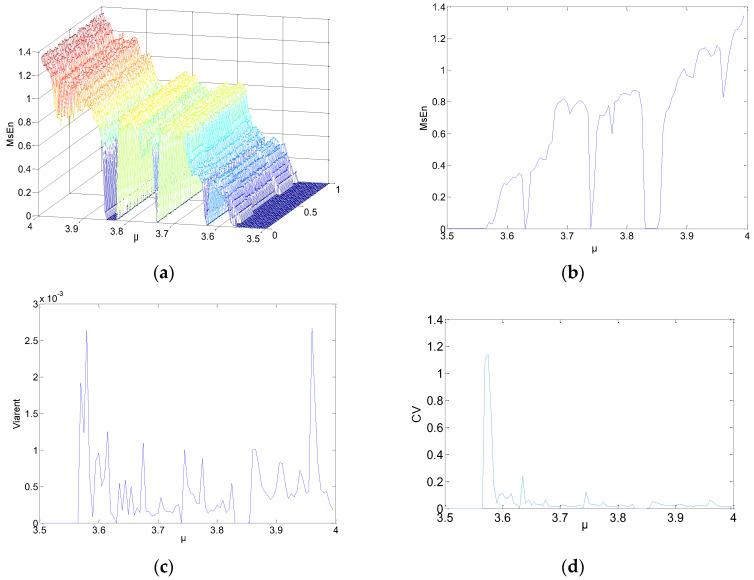
MSE results: (**a**) 3D figure of MSE; (**b**) means of MSE; (**c**) variances of MSE; (**d**) coefficient of variable of a different *μ*.

Substantial experiments with different parameters were carried out in order to obtain extensive results. More representative results were selected and are shown in Figure 1, Figure 2 and Figure 3. *N* = 1000, 2000, and 5000 were chosen as the lengths for the chaotic sequences. Set the parameters of MSE as *m* = 2, and *r* = 0.15. The scale factor was *τ* = 2. The initial values were $x_{0}\in \left(0,\;1\right)$. The step was 0.01; 100 points were utilized. The parameters were μ∈(3.5, 4), and the step was 0.005. Calculate the MSE of the logistic chaotic time series. Figure 1 shows the analysis results of MSE for the time series *N* = 1000, where Figure 1a is a 3D figure of the MSE results, Figure 1b is the means of different MSE values when *μ* is fixed, Figure 1c shows the variances of the MSE values, and Figure 1d is the coefficient variables of a different *μ*.

As shown in Figure 1a–Figure 3a, when *μ* is fixed, regardless of the data length or the initial value of the system, the MSE values of the logistic system remain unchanged. When *μ* increases, MSE increases as well. For a specific *μ*, some mutational points may show up when the entropy increases, and the logistic system maintains a bifurcated rather than chaotic status. As a result, the MSE values of the mutational points approach *μ* < 3.57 or even lower. Some information is summarized, as follows:

1. The 3D graph in Figure 3a appears smoother than Figure 1a and Figure 2a. In the same statistic conditions and threshold conditions, time series with longer lengths indicate a more stable statistical probability and lower degrees of dispersion. Hence, the length of the sequences has an impact on MSE.
2. In Figure 1c,d, the MSE value changes significantly. Because the length of the sequence is reduced by half during the process of coarse-graining. For the MSE method, although the number of points were reduced, the coarse-graining time series is not a subset of the original time series. On the contrary, the series includes all of the information about the original time series. Therefore, the error caused by the reduction of length of the coarse-graining time series is likely to be lower than that caused by the subset of the original time series. That is to say, coarse-graining time series on large time scales is likely more irregular (and is assigned a higher MSE value) than the original time series.
3. Figure 2c,d indicate the variance and coefficient variables of a sequence at *N* = 2000. The mean value of the variances, excluding the maximum and minimum values, is about 0.0012. There are 23 coefficient of variable (CV) that exceed 5%, including the value near the parameter *μ* = 3.57, and the sequence is not in a completely chaotic status. Figure 3c,d indicate the variances and coefficient variables of sequences at *N* = 5000. The maximum variance is $2.5\times 10^{-3}$. The remaining points are not more than $0.1\times 10^{-3}$, other than the points near μ=3.57. The range of coefficient variables is from 1% to 3%. In other words, the changes of the initial value do not change the MSE values of the series. Not all chaotic characters of the logistic system have dependency on the initial value. For this, it is possible to make a reasonable estimation and prediction on a chaotic system from macroscopic perspectives.

## 5. The Parameter Estimation of Chaotic Sequences

This section explains the mean variance method based on statistical analysis theory. Then, it is applied on a logistic chaotic sequence and tent chaotic sequence.

### 5.1. The Parameter Estimation of the Logistic Chaotic Sequence

In the field of secure communication, the sensitivity to the initial value is an important feature of the logistic chaotic sequence cipher. The initial value (*x*_0_) and parameter (*μ*) are two important keys of sequence cryptography. Dubois [30] presented the exact closed solution when researching the recurrent generation of logistic chaos maps. It was indicated that the solution of the logistic growth model is not unpredictable, which is consistent with the experimental results mentioned in Section 4.

For a logistic chaotic cipher sequence with an adequate length, the key space of the chaotic sequence cipher can be reduced by taking the current iteration value of *x* as a key, without considering the initial value (*x*_0_), as long as the reasonable value of parameter (*μ*) is estimated.

The method of estimating parameter *μ* is based on the theory of statistical analysis. The variance mean method is used to obtain parameters uniformly in each interval within the range of the initial value and the parameter *μ*, and to generate chaotic sequences according to logistic chaotic mapping. First, a data table based on the mean of variances is generated. When estimating the parameters of a logistic sequence, the variance of the sequence can be obtained, and then the value range of parameter *μ* can be estimated roughly by looking at the table. The calculation process is as follows.

**Figure 4:**
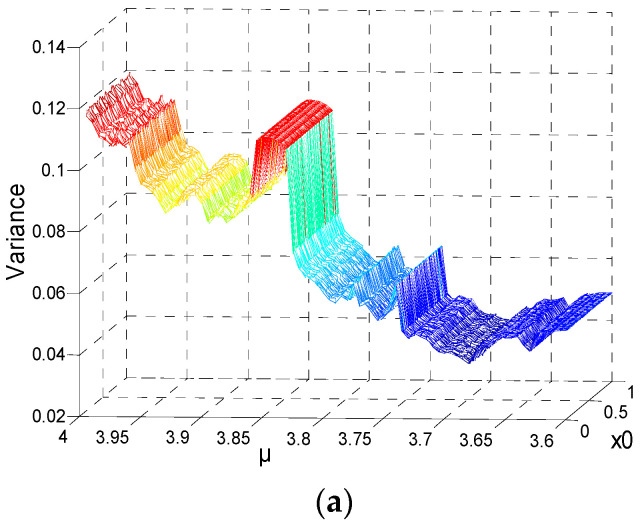

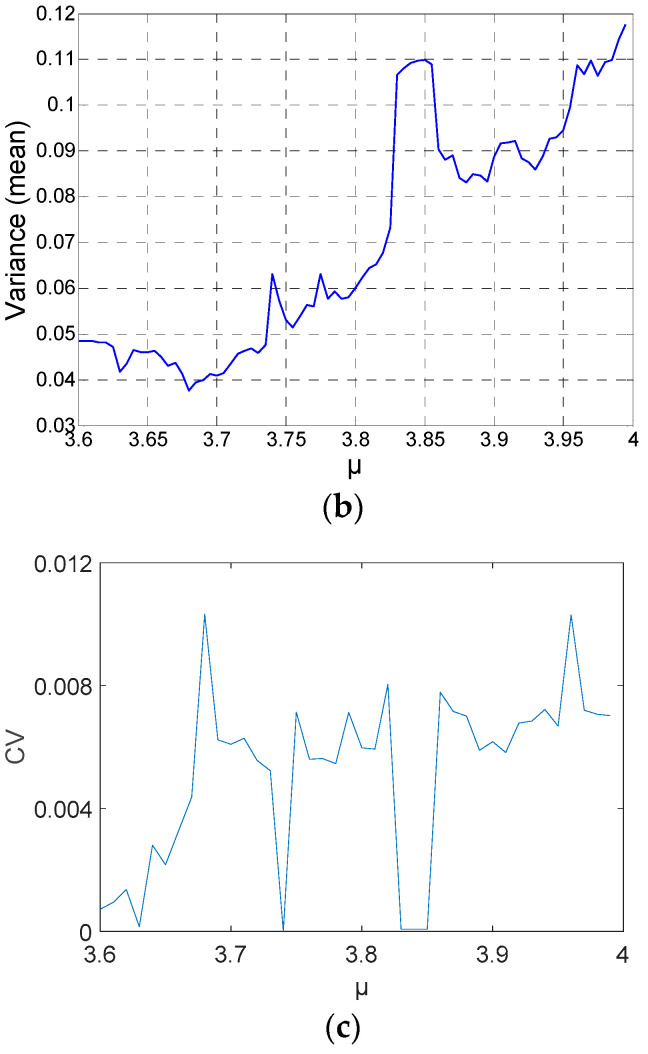
Results of variance analysis for *N* = 10^4^: (**a**) 3D figure of variance with different initial series; (**b**) the means of variances for different initial values; (**c**) CV of a different *μ*.

The initial values range $x_{0}\in \left[0,\;1\right]$, and the step is 0.01. The parameter is μ∈[3.6, 4.0], the step is 0.05, the length *N* = 10,000, and the variances of the series are shown in Figure 4. The formulation of variance is $Var\left(r\right)=\frac{\sum_{i=1}^{n}x_{i}-\mu }{n},\;\mathrm{and}\;\mu \in \left[3.6,\;4.0\right]$. Figure 4a indicates that different initial values for the same parameter (*μ*) have no impacts on variance distribution. Figure 4b shows the relationship between the means of variances and parameter *μ*. Figure 4c indicates that the maximum coefficient variable is not greater than 1%. Considering the relationship between the initial value and MSE value, the parameter *μ* of the series can be estimated when the logistic sequence has an adequate length. For the chaotic series, the key space of the time series password can be reduced.

**Figure 5:**
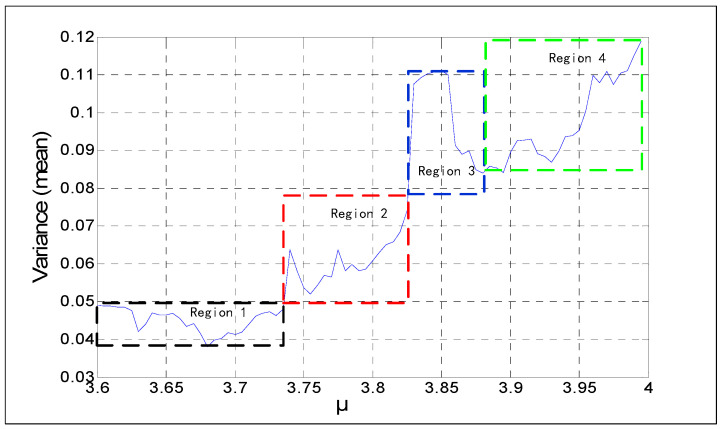
Initial value analysis of parameter *μ*.

According to the previous study, the initial value *x*_0_ does not impact the MSE values of a chaotic series. If a chaotic series has adequate length, the current value of *x* is a key. Parameter *μ* is the only key involved. Figure 4b divided into four regions is shown in Figure 5. The relations of the four sections are arranged in Table 1, which are the variances and *μ*. The range of the parameter *μ* can be obtained by calculating the variance of a sufficiently long chaotic sequence. Despite the fact that the exact value of the parameter cannot be obtained, it reduces the key space of the sequence to some extent. When the variance of the sequence belongs to Regions 1 or 2, the corresponding range of *μ* is unique and does not overlap with the other. But if the variance is in Regions 3 or 4, because there are some overlaps in the two regions, the parameter *μ* is harder to determine. The specific variance of the corresponding parameter *μ* is shown in Table 1.

**Figure 6:**
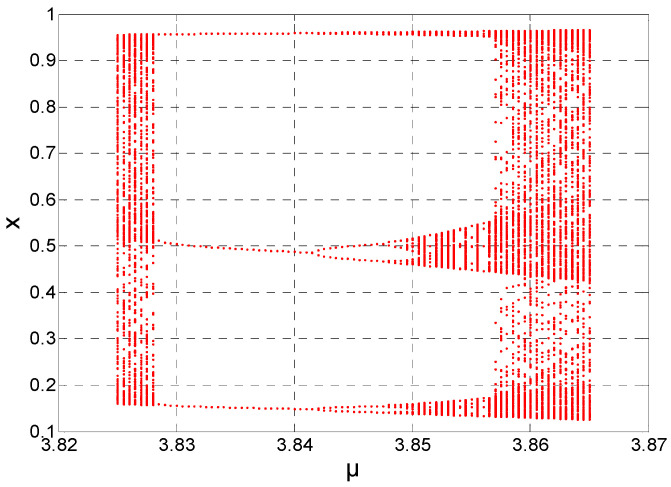
At *μ* = 3.825–3.865, logistic chaotic mapping.

**Figure 7:**
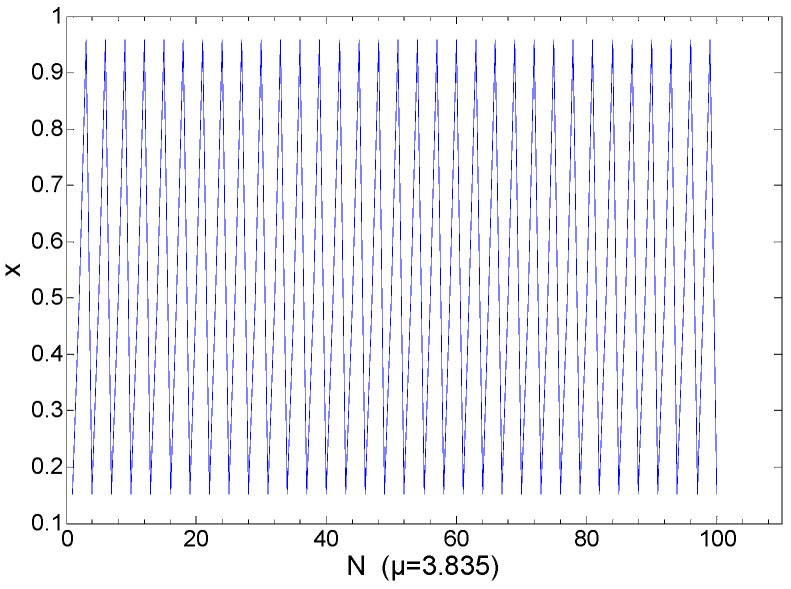
At *μ* = 3.835, logistic chaotic mapping.

**Figure 8:**
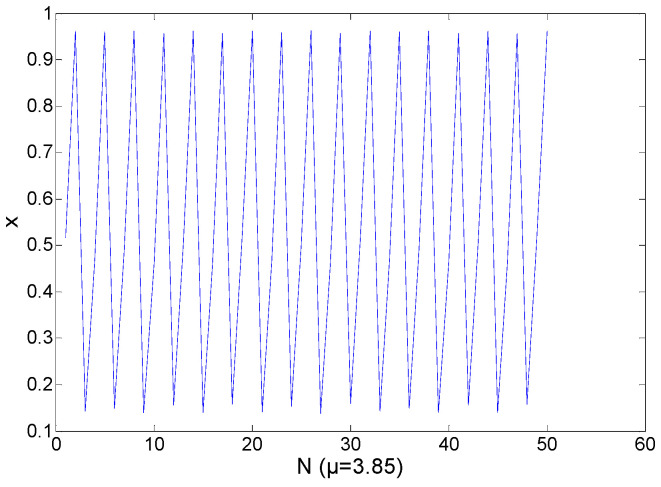
At *μ* = 3.85, logistic chaotic mapping.

In Region 3, the range of *μ* is from 3.825 to 3.865. As shown in Figure 6, in this region, the chaotic sequences are periodical, while in other regions they are opposite. Figure 7 shows the logistic chaotic mapping at *μ* = 3.835. Figure 8 shows the logistic mapping at *μ* = 3.85. In general, the parameter *μ* of Region 3 is not selected, because of its periodical. If the variance of a sequence is in Regions 3 or 4, Region 4 will have a higher probability.

### 5.2. The Parameter Estimated of the Tent Chaotic Sequence

**Figure 9:**
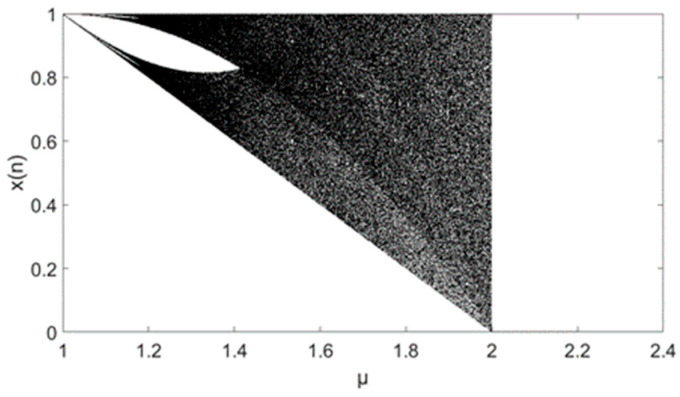
Tent chaotic mapping

**Figure 10:**
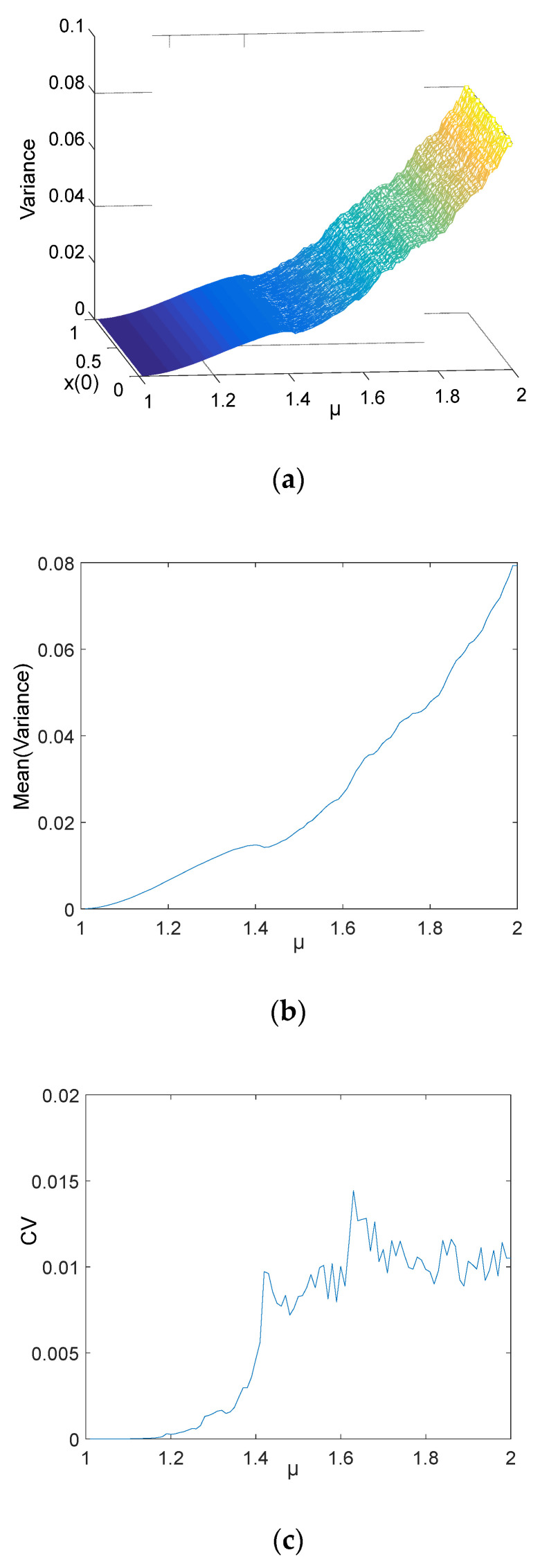
Results of the tent chaotic sequence: (**a**) three-dimension distribution of variance; (**b**) the means distribution of variance; (**c**) CV of a different *μ*.

The tent chaotic map is also one of the methods used to generate chaotic sequences in secure communication. As the topologically conjugate of the logistic, the sequence is also applied to the variance mean estimation method. The tent chaotic system is followed by Equation (10), and its system mapping is shown in Figure 9.

(10) $$x_{n+1}=1-\left|1-\mu x_{n}\right|\;\;\;\;\;\;x\in \left[0,\;1\right]$$

where $x_{0}\in \left[0,1\right]$ and parameter μ∈[1.0, 2.0]. After $10^{3}$ iterations, 10,000 chaotic sequences with a length of $10^{4}$ were selected. The three-dimensional distribution of the variance of each sequence is shown in Figure 10a. The formula for calculating the variance of different parameters is $\mathrm{Var}\left(r\right)=\frac{\sum_{i=1}^{n}x_{i}-\mu }{n}\;\mathrm{and}\;\mu =\in \left[1.0,\;2.0\right]$. In the three-dimensional distribution of variance, the CV of the variances for the same parameter *μ* are no more than 1.4%, which is shown in Figure 10c. That is, the chaotic sequence generated by the tent is not dependent on the initial value, and the sequence is more dependent on parameter *μ*. Table 2 was generated based on Figure 10b. It is an important reference to estimate the parameter *μ* of the tent chaotic sequence. There are five regions in Table 2, *μ* will be estimated when the variance of a tent chaotic sequence belongs to one of the five regions.

## 6. Conclusions

In this paper, through statistical theoretical analysis and mathematical simulation experiments, the MSE values of chaotic sequences under all of the available parameters are calculated. It is found that the complexity of the logistic chaotic sequences tends to be uniform and the density of periodic points is relatively weakened with the continuous increase of the logistic chaotic sequences. The initial value does not change the entropy value of the sequence too much, so not all of the characteristic parameters of the chaotic system have initial value dependence. As the chaotic sequence satisfies such a distribution characteristic, it is possible to make a reasonable estimation and prediction of the chaotic system from a macroscopic perspective. Considering that the security algorithm is public, only the key is secret, that is, the key involved in the chaotic sequence cryptography system is the initial value and parameter *μ* of the chaotic map. The statistical analysis and the variance mean method can reduce the key space and even achieve breakthroughs within a short period of time. Moreover, some system security engineers and mathematical cryptographers indeed give consideration to this influence when new solutions are proposed. The logistic chaotic system and the tent chaotic system were both verified in this paper, which proved that this method was effective.

## Figures and Tables

**Table 1:** Different regions and the corresponding variance.

Region	*μ*	Variance
Section 1	3.600–3.735	0.03805–0.04899
Section 2	3.735–3.825	0.04899–0.07406
Section 3	3.825–3.865	0.07406–0.11110
Section 4	3.865–3.995	0.08394–0.11880

**Table 2:** Five regions of the parameter *μ* and the corresponding variance.

Region	*μ*	Variance
1	1.00–1.19	2.451 × 10^−5^–0.00605
2	1.20–1.39	0.00657–0.01464
3	1.40–1.59	0.01476–0.02533
4	1.60–1.79	0.02652–0.04637
5	1.80–1.99	0.04774–0.07934
